# Efficacy, Safety, and Hematologic Recovery Following Intravenous Ferric Carboxymaltose in Patients with Iron Malabsorption-Related Iron Deficiency Anemia: A Prospective Clinical Study

**DOI:** 10.3390/nu18111807

**Published:** 2026-06-04

**Authors:** Silvia Scalamonti, Giulia Pivetta, Francesco Paolo Schiavone, Micaela Magnante, Manuela Pompili, Marica Vavallo, Bruno Annibale, Edith Lahner

**Affiliations:** Digestive Disease Unit, Department of Medical-Surgical Sciences and Translational Medicine, Sant’Andrea Teaching Hospital, Sapienza University of Rome, Via di Grottarossa 1035, 00189 Rome, Italy; silvia.scalamonti@uniroma1.it (S.S.); bruno.annibale@uniroma1.it (B.A.)

**Keywords:** iron deficiency anemia, ferric carboxymaltose, intravenous iron therapy, gastrointestinal iron malabsorption, autoimmune gastritis, celiac disease

## Abstract

**Background/Objectives**: Autoimmune gastritis (AIG), celiac disease (CD), and gastric surgery (GS) often cause iron deficiency anemia (IDA) due to iron malabsorption. In this clinical context, IDA treatment is often challenging. The first-line IDA treatment is oral iron supplementation followed by intravenous (IV) iron administration when ineffective or not tolerated. Ferric carboxymaltose (FCM) showed efficacy in various clinical settings. Prospective data evaluating the efficacy of IV FCM in IDA patients secondary to iron malabsorption are scant. The aim of the current study was to assess the tolerability, efficacy, and QoL impact of IV FCM for the treatment of IDA patients with iron malabsorption. **Methods**: Study design: single-center, prospective observational study: n = 37 adults with AIG, CD, or GS with IDA receiving IV FCM were consecutively included. Endpoints were (i) safety tolerability, (ii) efficacy on IDA recovery (Hb normalization), and (iii) QoL impact. At baseline (T0) and 12 weeks after treatment (T12), a QoL-SF12 questionnaire was assessed. Complete blood count (CBC) and iron status (ferritin, iron, transferrin, transferrin saturation (TS)) were assessed at T0, 4 weeks (T4), and T12 after treatment. **Results**: Of the 37 IDA patients, 19 (51.4%) had AIG, 9 (24.3%) CD, and 9 (24.3%) GS; Based on Ganzoni’s formula, 24 (64.9%) patients received a single IV FCM infusion (mean ± SEM dosage of 975 ± 12 mg); 13 (35.1%) required two IV infusion sessions with a mean ± SEM cumulative dose of 1400 ± 77 mg. One patient (2.7%) experienced mild adverse events without need for treatment interruption or hospitalization. At T0, anemia was moderate in 7 (18.9%) patients and severe in 1 (2.7%). IDA recovery was achieved in 26 (70.3%) patients at T4 and in 29 (78.4%) at T12. At T4, mean ± SEM Hb increased from 10.8 ± 0.2 g/dL to 12.7 ± 0.1 g/dL, ferritin from 28.5 ± 11.2 ng/mL to 188.2 ± 25.7 ng/mL, and TS from 6.7 ± 0.5% to 23.7 ± 1.9% (*p* < 0.0001). At T12, mean ± SEM Hb further increased to 13.1 ± 0.2 g/dL (*p* < 0.05 vs. T4), ferritin slightly decreased to 125 ± 26.7 ng/mL, and TS to 22.7 ± 2.8%. At T12, nonsignificant increases in QoL scores relative to baseline were observed. **Conclusions**: IV FCM is a safe and effective treatment leading to IDA recovery in nearly 80% of patients at T12. Thus, when oral iron treatment is not feasible or has failed, IV FCM treatment might be considered a first-line therapeutic option for IDA consequent to iron malabsorption.

## 1. Introduction

Iron deficiency (ID) is the most prevalent nutritional disorder globally, and its progression to iron deficiency anemia (IDA) represents a significant public health challenge, affecting about two billion people worldwide [1,2,3]. Far from being a mere hematological abnormality, IDA is a systemic condition with profound clinical consequences that significantly impair patient well-being and functionality [4]. Individuals with IDA may be asymptomatic or experience fatigue, irritability, depression, difficulty in concentrating, restless legs syndrome, pica, an eating disorder characterized by the psychologically compulsive craving or consumption of objects that are not normally consumed, dyspnea, lightheadedness, exercise intolerance, and heart failure. Symptom prevalence varies depending on age, comorbidities (e.g., chronic kidney or heart disease), and severity and rate of development of iron deficiency [5].

It is well known that IDA significantly affects quality of life (QoL), and recent evidence shows that IDA treatment improves QoL regardless of the underlying cause for anemia [6,7,8,9]. The maintenance of iron homeostasis is a tightly regulated process, with the primary site of absorption located in the duodenum and proximal jejunum. Dietary iron exists in two forms: heme iron, derived from hemoglobin and myoglobin, and non-heme iron (Fe^3+^), found in plant-based foods. The absorption of non-heme iron, which constitutes the majority of dietary intake, is a multi-step process highly dependent on the gastric and duodenal microenvironments. Gastric acid (HCl) plays a critical role by solubilizing ferric (Fe^3+^) iron salts and facilitating their reduction to the more soluble and readily absorbable ferrous (Fe^2+^) state. This conversion is enzymatically mediated at the apical membrane of enterocytes by duodenal cytochrome b. Subsequently, the divalent metal transporter 1 transports Fe^2+^ into the cell. Any disruption to this intricate pathway, anatomical, inflammatory, or pH-dependent, can lead to iron malabsorption and the eventual depletion of systemic iron stores, culminating in IDA [10,11].

The most common causes of ID are gastrointestinal or menstrual bleeding, impaired iron absorption, inadequate dietary iron intake, and pregnancy [5]. In particular, several gastrointestinal disorders may cause chronic iron malabsorption, and in patients affected by these disorders, IDA is often refractory to standard treatment [12]. One frequent and often misrecognized cause of iron malabsorption is autoimmune gastritis at its atrophic stage involving the corpus mucosa (AIG) [13,14]. AIG is a preneoplastic condition characterized by the destruction of parietal cells within the corpus oxyntic mucosa, leading to hypochlorhydria or, in the most severe cases, to achlorhydria. The lack of gastric acid directly impairs the reduction of dietary Fe^3+^ to the absorbable Fe^2+^ form, creating a powerful and persistent barrier to iron absorption, even in the presence of a structurally intact small intestine [11,13,14,15]. Another common cause of iron malabsorption is celiac disease (CD), an immune-mediated enteropathy triggered by gluten ingestion in genetically susceptible individuals. The resultant chronic inflammation leads to progressive villous atrophy, crypt hyperplasia, and a flattening of the small intestinal mucosa. This architectural damage severely reduces the surface area available for nutrient absorption, precisely in the duodenum and jejunum, the primary sites for iron uptake. Notably, IDA is one of the most common extra-intestinal manifestations of CD and can, in many cases, be its sole presenting feature [16,17,18]. Gastric surgery (GS), including total or partial gastrectomy for malignancy and bariatric procedures such as the Roux-en-Y gastric bypass, frequently results in iatrogenic iron malabsorption. The mechanisms are multifactorial and include reduced gastric acid secretion, rapid gastric emptying, and, most critically, the surgical bypass of the duodenum and proximal jejunum, which excludes the primary absorptive surfaces from contact with ingested nutrients [19,20,21].

The standard first-line treatment for IDA is oral iron supplementation. However, in the context of the aforementioned malabsorptive syndromes, this approach is fraught with challenges that limit its clinical utility, such as a lack of efficacy, gastrointestinal side effects and poor adherence [22]. When oral iron supplementation is ineffective, not tolerated, or clinically inappropriate, intravenous (IV) iron administration becomes the treatment of choice. By delivering iron directly into the circulation, IV formulations bypass the compromised gastrointestinal tract entirely, ensuring complete bioavailability for erythropoiesis and replenishment of iron stores in the reticuloendothelial system. Modern IV iron preparations have evolved significantly from older dextran-based compounds, offering improved safety profiles [23]. Ferric carboxymaltose (FCM) is a stable, non-dextran, macromolecular ferric hydroxide carbohydrate complex that permits the administration of large replacement doses (e.g., 1000 mg of elemental iron) in a single, rapid infusion of approximately 15 min. Its efficacy and safety have been established in various clinical settings, including chronic kidney disease, heart failure, and inflammatory bowel disease, where it has been shown to rapidly correct anemia, replete iron stores, and improve quality of life [24]. A previous retrospective study showed that FCM was safe and effective for treating IDA associated with corpus atrophic gastritis, resulting in rapid and durable recovery [25]. Despite the strong theoretical rationale for using IV iron in IDA patients due to iron malabsorption [26], prospective data evaluating the efficacy and safety of FCM in this setting are lacking. Thus, the gap in prospective data on this topic leads to therapeutic difficulty in clinical practice.

Based on this background, the objectives of the current study were to assess the treatment efficacy in recovering from IDA, the tolerability, and the impact on QoL of IV FCM for the treatment of IDA in patients with established gastrointestinal causes of iron malabsorption.

## 2. Materials and Methods

This paper was drafted in accordance with the Strengthening the Reporting of Observational Studies in Epidemiology (STROBE) guidelines [27].

### 2.1. Study Design

This is a single-center, prospective observational study conducted in an academic teaching hospital, a referral center for gastric autoimmunity and micronutrient malabsorption. Adult patients (>18 years) with a previous diagnosis of AIG, CD, or a prior history of GS, along with a diagnosis of IDA refractory to oral iron treatment, receiving IV FCM treatment between December 2023 and December 2024 were consecutively included. Patients were defined as “refractory to oral iron treatment” when they were intolerant to oral iron treatment with troublesome side effects leading to treatment withdrawal or poor adherence, irrespective of the type of oral iron used, or when oral treatment did not lead to an increase in hemoglobin and iron parameters after at least three months of treatment. Patients receiving FCM treatment for other causes of IDA such as acute or chronic gastrointestinal bleeding; cardiological, pneumological, nephrological, gynecological, or neurological comorbidities; coagulation diseases; and/or trauma were excluded. Baseline vitamin B_12_ and folate status were assessed, and, when low, corrected with supplementation. Also, serum markers for acute inflammation were assessed to exclude anemia of chronic disease. Patients with CD were regularly monitored for adherence to a gluten-free diet by Biagi score [16,28].

For women, menstrual bleeding was accurately evaluated using a validated pictorial bleeding chart (Higham score: normal value < 160). Women with increased menstrual losses were excluded from the study. Patients were also excluded when biochemical or clinical follow-up was incomplete.

The endpoints of the current study were to assess (i) the safety and tolerability of IV FCM, (ii) its efficacy on IDA recovery (normalization of Hb levels), and (iii) its impact on QoL. At baseline (T0), patients were clinically evaluated to assess for IDA-related symptoms and their severity by using a structured questionnaire [29]. Furthermore, health and wellness perceptions were assessed at baseline (T0) and after 12 weeks of treatment (T12) using a validated QoL-SF12 questionnaire. This latter questionnaire produces two summary measures of self-perceived physical and mental health. Physical component summary (PCS) and mental component summary (MCS) scores were considered as an expression of good QoL when >44 and >46, respectively [30,31]. Complete blood count (CBC) and iron status (ferritin, iron, transferrin, transferrin saturation (TS)) were assessed.

The FCM dosage was calculated for each patient using Ganzoni’s formula [32]. After FCM IV treatment, CBC and iron status were again assessed at 4 (T4) and 12 (T12) weeks. The Qol SF12 questionnaire was repeated after 12 weeks.

Written informed consent was obtained from all participants, and approval of the local ethical committee was achieved (No. CE 7004_2020, 7 July 2020, Sapienza University Ethical Committee).

### 2.2. Diagnostic Criteria for IDA

IDA was diagnosed when Hb was <13 g/dL in men and <12 g/dL in women, ferritin was <45 ng/mL, and TS was <15% [18]. TS was calculated as the ratio of serum iron to the total iron-binding capacity of available transferrin. In particular, moderate and severe anemia were defined when Hb was 8–10 g/dL and Hb < 8 g/dL, respectively [1,5,12]. Red cell distribution width (%), a marker of anisocytosis, was also recorded (normal value < 14.6%).

The IV FCM treatment was considered efficacious when Hb levels (Hb > 13 g/dL for men and Hb > 12 g/dL for women) normalized at follow-up (T4 and/or T12).

### 2.3. Diagnostic Criteria for AIG, CD, and GS

AIG was defined by histopathology assessed on gastric biopsies taken during upper gastrointestinal endoscopy according to the updated Sydney system [33], and diagnosed when gastric mucosal atrophy with or without intestinal metaplasia was detected in the corpus mucosa along with a spared antral mucosa [13,14]. The eventual presence of *Helicobacter pylori* infection was also assessed by histopathology, and, when present, eradication treatment was prescribed.

CD was defined by histology on duodenal biopsies taken during upper gastrointestinal endoscopy according to the Marsh classification system modified by Oberhuber [28,34,35]: the presence of normal villous architecture and >30 intraepithelial lymphocyte/100 enterocytes was defined as Marsh I; the presence of normal villous architecture with >30 intraepithelial lymphocytes/100 enterocytes and crypt hyperplasia was defined as Marsh II; and the presence of both crypt hyperplasia and intraepithelial lymphocytosis associated with villous atrophy was defined as Marsh III, distinguished as A, B or C for mild, moderate or severe atrophy, respectively; along with positivity against anti-transglutaminase and/or anti-endomysial IgA autoantibodies [36].

GS as the cause of IDA: virtually all gastric surgical procedures may over time lead to micronutrient deficiencies, including total or partial gastric resection according to Billroth I or II performed for surgical treatment of gastric neoplasias or complicated and non-endoscopically treatable peptic ulcer, and bariatric surgical procedures performed for voluntary weight loss, such as sleeve gastrectomy, Roux-en-Y gastric bypass, or biliopancreatic diversion [20,21,37].

### 2.4. FCM Dosage Calculation and IV Treatment

FCM dosage was calculated as total iron deficit according to Ganzoni’s formula considering hemoglobin (Hb, g/dL) values and body weight (kg): [total iron deficit, mg = weight, kg × (target Hb, g/dL − actual Hb, g/dL) × 2.4 + iron stores, mg], evaluating the iron requirements of each patient [30]. If the required dosage was up to 1000 mg, an IV infusion was scheduled in a single session; for a dosage of more than 1000 mg, a second infusion after 1 week was scheduled.

As previously reported [25], the FCM preparation was diluted in 250 mL of sterile sodium chloride 9 mg/mL (0.9%) solution for IV infusion and administered slowly over 30 min. This slow infusion was chosen to minimize the risk of adverse effects, as patients were treated in an outpatient setting. During and after the infusion, the patients were carefully monitored by a specialized nurse for at least 30 min, and any adverse event was promptly assessed for the need to discontinue the IV infusion or to initiate specific medical therapy.

### 2.5. Statistical Analyses

Descriptive statistics were performed using mean ± SEM, and median (range) for quantitative variables. Percentages of total number were computed for qualitative variables. Student’s *t*-test for paired samples was used to compare Hb and iron status parameters at baseline (T0) to those at T4 and T12 after IV FCM treatment. The chi-square test was used to compare qualitative (dichotomous) variables. A *p*-value of less than 0.05 was considered statistically significant. Statistical analyses were performed with MedCalc Statistical Software version 22.009 (MedCalc Software, Ostend, Belgium; http://www.medcalc.org; 2023).

## 3. Results

### 3.1. Study Population

Overall, 37 patients with IDA were included. IDA was secondary to AIG in 19 (51.4%), CD in nine (24.3%), and GS in a further nine (24.3%) patients.

The female gender was slightly preponderant (n = 23, 62.2%). The median age was 70 years (range 32–90). At baseline, IDA-related symptoms were present in all 37 patients, and their distribution and severity (mild, moderate, severe) are shown in Table 1.

Among the 19 AIG patients (females, n = 9, 47.4%), *Helicobacter pylori* infection was successfully treated in five (26.3%) patients. Corpus atrophy was severe in 12 (63.1%) patients and associated with intestinal metaplasia in seven (36.8%) patients. Among the nine CD patients (females n = 8, 88.9%), all had duodenal villous atrophy (Marsh III) and were on a gluten-free diet. Among the nine GS patients (females n = 6, 66.7%), total (n = 1) or partial (n = 8) gastric resection was performed for neoplasia in two patients, for peptic ulcers in four patients, and for bariatric surgery in three patients. All 37 patients completed the 12-week follow-up.

### 3.2. IV FCM Treatment

Based on the FCM dosage calculation by Ganzoni’s formula, 24 (64.9%) patients received a single IV FCM infusion with a mean ± SEM dosage of 975 ± 12 mg. The remaining 13 (35.1%) patients exceeded the iron need of 1000 mg and thus required two sessions of IV infusion, with a mean ± SEM cumulative dose of 1400 ± 77 mg. No additional infusions between T4 and T12 were required.

### 3.3. Tolerability of IV FCM Treatment

Overall, IV FCM was well tolerated, and all 37 patients completed the IV infusion of the calculated FCM dosage. Only one patient (2.7%) experienced mild adverse events (itching and cutaneous erythema), which were resolved after administration of IV hydrocortisone and chlorpheniramine maleate, without the need for FCM treatment interruption or hospitalization. No moderate or severe adverse reactions, late-onset events, or deaths were reported.

### 3.4. Efficacy of IV FCM Treatment on Anemia and Iron Status Recovery

At baseline (T0), anemia was moderate in seven (18.9%) and severe in one (2.7%) patient, while 29 (78.4%) patients had mild anemia. As shown in Table 2, at T0, the mean ± SEM hemoglobin (Hb) level was 10.8 ± 0.2 g/dL, the mean ± SEM serum ferritin was 28.5 ± 11.2 ng/mL, the mean transferrin saturation (TS) ±SEM was 6.7 ± 0.5%, and the mean ± SEM RDW was 15.4 ± 0.5%.

Recovery from IDA, defined as normalization of Hb levels, was achieved in 26 (70.3%) patients at T4 and in 29 (78.4%) at T12.

At week 4 (T4) after IV FCM treatment, mean ± SEM Hb increased to 12.7 ± 0.1 g/dL, mean ± SEM ferritin to 188.2 ± 25.7 ng/mL, and mean ± SEM TS to 23.7 ± 1.9%. All these changes were statistically significant (*p* ≤ 0.0001), corresponding to an increase of 2 g/dL for Hb, >100 ng/mL for ferritin, and 17 percentage points for TS, respectively. At 12 weeks (T12) after IV FCM treatment, mean ± SEM Hb further increased to 13.1 ± 0.2 g/dL (*p* < 0.05 vs. T4), while mean ± SEM ferritin slightly decreased to 125 ± 26.7 ng/mL, and mean ± SEM TS to 22.7 ± 2.8%. Mean ± SEM RDW increased significantly at T4 (18.6 ± 0.7% vs. 15.4 ± 0.5% at T0; *p* = 0.0003), while a significant decrease at T12 was reported (versus T4 14.8 ± 0.4%; *p* < 0.0001). The continuous upward trend in Hb remained statistically significant, whereas ferritin and TS showed a slight, nonsignificant decrease compared with T4, and RDW decreased to levels similar to baseline (Table 2). The temporal profile of Hb showed a rapid improvement at T4 and a more gradual increase at T12, with an overall gain of approximately 3 g/dL from baseline. Ferritin and TS displayed a marked rise at T4, followed by modest reductions and the achievement of a plateau at T12, while RDW displayed a marked rise at T4, too, but a significant decrease at T12.

The response to IV FCM treatment was substantially similar in the three etiology groups of IDA, autoimmune gastritis, celiac disease, and gastric surgery, and comparable to the response of the pooled patients. Table 3 shows the hematological and iron status parameters at baseline (T0) and at 4 and 12 weeks after IV FCM infusion.

### 3.5. Impact of IV FCM Treatment on QoL

At baseline, the mean ± SEM PCS score was 39.3 ± 1.5 (median 39.6, range 20.5–56.6), and the mean ± SEM MCS score was 42.0 ± 1.7 (median 41.6, range 25.3–58.6). At T12, slight, nonsignificant increases were observed (PCS: mean ± SEM 39.5 ± 1.5; median 41.28, range 20.3–56, and MCS: mean ± SEM 44.6 ± 1.3, median 45.75, range 26.7–58.6).

The proportion of patients exceeding the cut-off for good QoL (PCS > 44, MCS > 46) slightly increased at T12: those with PCS > 44 increased from 11 (29.7%) at T0 to 13 (35.1%) at T12, and patients with MCS > 46 increased from 14 (37.8%) at T0 to 18 (48.6%) at T12, without reaching statistical significance (*p* > 0.05).

## 4. Discussion

To the best of our knowledge, this is the first prospective observational study investigating the safety and efficacy of IV FCM in patients with persistent IDA refractory to oral iron treatment, caused by chronic iron malabsorption due to carefully diagnosed chronic conditions of the upper gastrointestinal tract, such as AIG, CD, or previous GS impairing iron absorption.

This study showed that IV FCM is a valid therapeutic option, leading to rapid and significant correction of hematological and biochemical parameters in patients with IDA consequent to iron malabsorption in whom oral iron treatment is not feasible or has failed. The mean increase in Hb of approximately 3 g/dL 12 weeks after treatment, along with the normalization of iron stores (ferritin and TS) and normalization of RDW, confirms the ability of IV FCM to effectively treat IDA patients with iron malabsorption due to the compromised gastrointestinal function. Notably, by 4 weeks after IV FCM treatment, 70% of treated patients had completely recovered from IDA as shown by normal Hb levels, and at 12 weeks after treatment, this figure increased to nearly 80%.

Ferritin levels and TS followed the rapid increase in Hb levels at 4 weeks, but after this time point, a plateau without further increase was reached. The evolution over time of iron stores shows first a rapid repletion to a maximum, which is then maintained until 12 weeks after iron treatment.

A similar result was observed in a previous retrospective study that included only patients with corpus atrophic gastritis, in which recovery from anemia was observed in 67% and 78% of patients at 4 and 12 weeks after treatment, respectively [25]. In contrast, in this previous study, at T12, ferritin levels significantly decreased again, suggesting an insufficient FCM dosage. Indeed, in this last study, the dosage was based on hemoglobin levels only, without applying Ganzoni’s formula, which also considers body weight. Thus, these results show that a correct dosage calculation leads to a more efficacious replenishment of iron stores, as shown by stable ferritin levels 12 weeks after treatment.

Interestingly, RDW, a well-known marker of anisocytosis that reflects heterogeneity in the size of circulating erythrocytes, also showed a rapid increase at four weeks after treatment, indicating rapid bone marrow activation and the entry of immature erythrocytes into the bloodstream, increasing RDW. This phenomenon was no longer present at 12 weeks after treatment, when bone marrow erythropoiesis became more regular and efficacious, with a normalized RDW [38].

The standard first-line treatment for IDA is oral iron supplementation. However, in the context of the aforementioned malabsorptive conditions, this approach is fraught with challenges that limit its clinical utility. The pathophysiology that causes anemia—be it mucosal oxyntic mucosa atrophy, achlorhydria, surgical resection or anatomical bypass—may also compromise the absorption of therapeutic oral iron, making it ineffective for replenishing iron stores. Oral iron preparations are notoriously associated with a high incidence of gastrointestinal side effects, including nausea, epigastric pain, constipation, or diarrhea. These adverse events severely undermine long-term patient compliance, often leading to treatment discontinuation and failure to achieve or maintain target Hb levels and satisfy iron stores. This creates a vicious circle of ineffective treatment, persistent symptoms, and poor patient outcomes [23,24,25].

The findings of the current study emphasize the use of a personalized therapeutic approach in terms of iron dosage for IDA in patients with malabsorption syndromes. The cornerstone of this approach was the use of Ganzoni’s formula to calculate each patient’s individual iron requirement precisely. One pitfall of this approach is that patients requiring more than 1000 mg of FCM must undergo a second IV infusion, as the maximum dose that can be administered in a single infusion is 1000 mg [7,25,39]. This second IV infusion session means an increased workload for specialized nurses and requires more appointments and IV infusion chairs. Nevertheless, the good therapeutic result at 4-week follow-up, and even more so at 12-week follow-up, overcomes this drawback.

In the current study, it was interesting to note that despite the relatively high proportion of patients with mild anemia (78.4%), all patients reported IDA-related symptoms; amongst the most commonly reported were sweating, dizziness, blurry vision, tinnitus, headache, chest pain, and palpitations, which were classified as severe in up to 27% of cases. This finding supports the view that IDA can be defined as a systemic condition with profound clinical consequences that significantly impair patient well-being and function [4]. In the current study, the overall QoL PCS and MCS score slightly increased at 12-week follow-up along with the absolute number of patients with good QoL, but without reaching statistical significance. This might be due to the relatively short follow-up for evaluating a direct effect on PCS and/or MCS, and to the fact that about one-third of patients showed normal PCS and MCS scores at baseline. On the other hand, it might be considered that these IDA patients are affected by chronic gastrointestinal diseases that cause iron malabsorption. For this reason they might be used to the impact of IDA on their QoL, thus experiencing a lower level of perception. Also, awareness that in the future iron stores may decrease again and new iron supplementation may be needed might have influenced the scoring of QoL of health and wellness perception. However, at least in a subgroup of these IDA patients, the benefits of the current treatment approach went beyond mere biochemical correction and led to perceived improvements in patients’ well-being.

Moreover, the current study confirmed a high safety profile of IV FCM, with only 1 of 37 patients experiencing a mild adverse effect that was resolved quickly without treatment interruption. This result provides reassurance about the use of modern IV iron formulations, which have overcome the safety limitations of older dextran-based compounds [40]. However, the tolerability in the current study refers mainly to clinically observed infusion reactions and short-term adverse events, not to a complete biochemical safety assessment.

The current study has several limitations that should be considered when interpreting the findings. First, this was a single-center prospective observational study with a relatively small sample size, which may limit the generalizability of the results and reduce statistical power for subgroup analyses. In particular, the inclusion of patients with different etiologies of gastrointestinal iron malabsorption, including AIG, CD, and GS, introduces clinical heterogeneity that may influence hematologic response and treatment outcomes, albeit our data did not show substantial differences in treatment response between groups. Another important limitation is the absence of a control group receiving oral iron supplementation, a placebo, or alternative intravenous iron formulations. Consequently, direct comparisons regarding efficacy, tolerability, and quality-of-life improvement could not be performed. In addition, the follow-up period was limited to 12 weeks, preventing assessment of long-term maintenance of iron stores, anemia relapse rates, retreatment requirements, and sustained clinical benefits over time. Although hematological and biochemical parameters significantly improved after treatment, clinical symptoms related to IDA were not reassessed during follow-up. Therefore, the laboratory response observed cannot be fully translated into objective clinical improvement in symptom burden or functional status. Similarly, QoL evaluation showed only nonsignificant changes, possibly due to the relatively short follow-up period, the chronic nature of the underlying gastrointestinal disorders, and the limited sample size. Another relevant limitation is the lack of serum phosphate monitoring. FCM has been associated with hypophosphatemia [41,42], particularly after repeated administration, and the current study therefore cannot provide information on the incidence or clinical relevance of this adverse effect in patients with gastrointestinal iron malabsorption. However, patients were clinically monitored and none of the patients reported new onset of symptoms possibly related to low serum phosphate levels, such as bone pain or muscle weakness. Finally, statistical analyses were primarily based on univariate comparisons and did not include multivariate or longitudinal modeling approaches that could better account for repeated measurements and potential confounding factors. Larger multi-center controlled studies with longer follow-up and standardized clinical assessments are warranted to further validate the efficacy, safety, and long-term therapeutic role of intravenous FCM in this patient population.

## 5. Conclusions

In conclusion, IV FCM is an effective and safe treatment, leading to recovery from IDA in nearly 80% of cases at 12 weeks after treatment, with maintenance of iron stores replenished. Thus, when oral iron treatment is not feasible or has failed, IV FCM treatment might be considered a first-line therapeutic option for patients with IDA consequent to gastrointestinal iron malabsorption.

## 6. Future Perspectives

1. Future studies should validate these findings in larger multi-center cohorts with longer follow-up to better assess the long-term efficacy, safety, and durability of IV FCM therapy in patients with gastrointestinal iron malabsorption.
2. Randomized controlled trials comparing FCM with oral iron and other intravenous formulations are needed to establish optimal therapeutic strategies for AIG, CD, and post-GS patients.
3. Additional investigations should also evaluate retreatment schedules, recurrence rates, symptom improvement, fatigue, and quality-of-life outcomes. Particular attention should be given to monitoring treatment-related adverse effects, especially hypophosphatemia associated with FCM administration.
4. Finally, future translational approaches focusing on personalized iron replacement protocols tailored to disease etiology, iron deficit, and patient characteristics may improve clinical management and long-term outcomes in this population.

## Figures and Tables

**Table 1:** Symptoms related to iron-deficiency anemia at baseline in the 37 patients with gastrointestinal iron malabsorption.

	Severity of Symptoms		
Type of Symptoms	Mild	Moderate	Severe
Dizziness	27 (73)	5 (13.5)	5 (13.5)
Blurry vision	26 (70.3)	7 (18.9)	4 (10.8)
Tinnitus	29 (78.4)	7 (18.9)	1 (2.7)
Headache	25 (67.6)	5 (13.5)	7 (18.9)
Fatigue	18 (48.6)	13 (35.1)	6 (16.2)
Sleeping disorders	20 (54.1)	9 (24.3)	8 (21.6)
Nervousness, crankiness	17 (45.9)	12 (32.4)	8 (21.6)
Sweating	30 (81.1)	6 (16.2)	1 (2.7)
Shortness of breath	12 (32.4)	15 (40.5)	10 (27.0)
Chest pain	25 (67.6)	8 (21.6)	4 (10.8)
Overfatigue	15 (40.5)	12 (32.4)	10 (27.0)
Difficulty in concentration	20 (54.1)	8 (21.6)	9 (24.3)
Depressive symptoms	22 (59.5)	9 (24.3)	6 (16.2)
Agitation	17 (45.9)	10 (27.0)	10 (27.0)
Oversensitivity, increased susceptibility	17 (45.9)	10 (27.0)	10 (27.0)
Poor cold tolerance	14 (37.8)	13 (35.1)	10 (27.0)
Restless legs syndrome	14 (37.8)	13 (35.1)	10 (27.0)
Palpitations	23 (62.2)	8 (21.6)	6 (16.2)

Data are expressed as number (%).

**Table 2:** Hematological (hemoglobin, red cell distribution width) and iron status parameters (ferritin, transferrin saturation) at baseline (T0) and at 4 and 12 weeks after intravenous ferric carboxymaltose infusion.

	Baseline (T0)	Week 4 (T4)	Week 12 (T12)
Hemoglobin (Hb, g/dL)	10.8 ± 0.2	12.7 ± 0.1 *	13.1 ± 0.2 °
Red cell distribution width (%)	15.4 ± 0.5	18.6 ± 0.7 ^x^	14.8 ± 0.4 *
Ferritin (ng/mL)	28.5 ± 11.2	188.2 ± 25.7 *	125.0 ± 26.7 *
Transferrin saturation (%)	6.7 ± 0.5	23.7 ± 1.9 *	22.7 ± 2.8 *

Data are shown as mean ± SEM. * *p* ≤ 0.0001 vs. T0; **°** *p* < 0.05 vs. T4; ^x^ *p* = 0.0003 vs. T0.

**Table 3:** Hematological (hemoglobin, red cell distribution width) and iron status parameters (ferritin, transferrin saturation) at baseline (T0) and at 4 and 12 weeks after intravenous ferric carboxymaltose infusion by etiology of iron deficiency anemia.

**Autoimmune Gastritis**	**Baseline (T0)**	**Week 4 (T4)**	**Week 12 (T12)**
Hemoglobin (Hb, g/dL)	10.5 ± 0.4	12.7 ± 0.4 *	13.0 ± 0.3 ^
Red cell distribution width (%)	15.6 ± 0.7	17.7 ± 1.0	14.4 ± 0.3
Ferritin (ng/mL)	29.2 ± 9.0	263.8 ± 45.5 *	159.4 ± 35.5 ^
Transferrin saturation (%)	7.9 ± 0.9	21.5 ± 2.7 *	23.4 ± 2.8 ^
**Celiac disease**	**Baseline (T0)**	**Week 4 (T4)**	**Week 12 (T12)**
Hemoglobin (Hb, g/dL)	11.1 ± 0.3	12.9 ± 0.3 **	13.2 ± 0.4 ^
Red cell distribution width (%)	14.9 ± 0.9	20.1 ± 0.8 **	14.4 ± 0.7
Ferritin (ng/mL)	4.8 ± 0.7	90.7 ± 9.4 *	68.5 ± 3.4 ^
Transferrin saturation (%)	4.6 ± 0.7	26.4 ± 4.0 *	24.8 ± 4.1 ^
**Gastric surgery**	**Baseline (T0)**	**Week 4 (T4)**	**Week 12 (T12)**
Hemoglobin (Hb, g/dL)	11.1 ± 0.3	12.6 ± 0.3 **	13.0 ± 0.4 ^^
Red cell distribution width (%)	15.1 ± 0.9	19.1 ± 1.3 **	15.9 ± 1.0
Ferritin (ng/mL)	48.0 ± 40.0	169.8 ± 30.3 **	142.7 ± 81.1 ^
Transferrin saturation (%)	6.4 ± 0.8	24.4 ± 4.2 *	19.1 ± 2.2 ^

Data are shown as mean ± SEM. T0 vs. T4 * *p* < 0.001; ** *p* < 0.01; ^ T0 vs. T12 *p* < 0.001; ^^ T0 vs. T12 *p* < 0.01.

## Data Availability

The raw data presented in this study will be made available by the corresponding author on request due to legal reasons.

## Abbreviations

The following abbreviations are used in this manuscript: AIG, autoimmune gastritis; CBC, complete blood cell count; CD, celiac disease; FCM, ferric carboxymaltose; GS, gastric surgery; ID, iron deficiency; IDA, iron deficiency anemia; IV, intravenous; Hb, hemoglobin; MSC, mental component summary; PCS, physical component summary; QoL, quality of life; SEM, standard error of mean; TS, transferrin saturation.
